# Deficiency of complement component 5 ameliorates glaucoma in DBA/2J mice

**DOI:** 10.1186/1742-2094-10-76

**Published:** 2013-06-27

**Authors:** Gareth R Howell, Ileana Soto, Margaret Ryan, Leah C Graham, Richard S Smith, Simon WM John

**Affiliations:** 1 The Jackson Laboratory, 600 Main Street, Bar Harbor, Maine, ME, USA; 2 Howard Hughes Medical Institute, 600 Main Street, Bar Harbor, ME, USA; 3 Department of Ophthalmology, Tufts University School of Medicine, Boston, MA, USA

## Abstract

**Background:**

Glaucoma is an age-related neurodegenerative disorder involving the loss of retinal ganglion cells (RGCs), which results in blindness. Studies in animal models have shown that activation of inflammatory processes occurs early in the disease. In particular, the complement cascade is activated very early in DBA/2J mice, a widely used mouse model of glaucoma. A comprehensive analysis of the role of the complement cascade in DBA/2J glaucoma has not been possible because DBA/2J mice are naturally deficient in complement component 5 (C5, also known as hemolytic complement, Hc), a key mediator of the downstream processes of the complement cascade, including the formation of the membrane attack complex.

**Methods:**

To assess the role of C5 in DBA/2J glaucoma, we backcrossed a functional *C5* gene from strain C57BL/6J to strain DBA/2J for at least 10 generations. The prevalence and severity of glaucoma was evaluated using ocular examinations, IOP measurements, and assessments of optic nerve damage and RGC degeneration. To understand how C5 affects glaucoma, C5 expression was assessed in the retinas and optic nerves of C5-sufficient DBA/2J mice, using immunofluorescence.

**Results:**

C5-sufficient DBA/2J mice developed a more severe glaucoma at an earlier age than standard DBA/2J mice, which are therefore protected by C5 deficiency. Components of the membrane attack complex were found to be deposited at sites of axonal injury in the optic nerve head and associated with RGC soma in the retina.

**Conclusion:**

C5 plays an important role in glaucoma, with its deficiency lessening disease severity. These results highlight the importance of fully understanding the role of the complement cascade in neurodegenerative diseases. Inhibiting C5 may be beneficial as a therapy for human glaucoma.

## Introduction

Glaucoma, the second leading cause of blindness worldwide, is a neurodegenerative disease involving the death of retinal ganglion cells (RGCs) and degeneration of the optic nerve [1-3]. Major risk factors include aging and increased intraocular pressure (IOP). A number of processes have been implicated in glaucoma, but the key molecules that mediate early damage remain unclear. Upregulation and activation of complement proteins are consistent features of human glaucoma and animal models of glaucoma [4-7]. Despite the importance of the complement cascade, the exact roles of specific pathway members in glaucoma have not been determined.

The complement cascade is activated as part of an innate immune response against pathogens. It also serves to clear unwanted cells and cellular debris [8], and has been shown to mediate synapse elimination in the central nervous system (CNS) [9]. Three major variants of the complement cascade have been widely studied: the classical, the alternative, and the lectin pathways. A major effector molecule of all three pathways is complement component C5 [10]. The C5 protein is cleaved by C5 convertases to produce C5a and C5b peptides. C5a is an anaphylotoxin that can mediate neuroinflammatory responses, while C5b is a key molecule in the formation of the membrane attack complex (MAC). The MAC is a transmembrane channel composed of the complement factors C5b, C6, C7, C8, and C9 (C5b-9) and is formed in the cell membrane of target cells, causing lysis [11].

DBA/2J mice are widely used as a model of inherited glaucoma. DBA/2J mice develop hallmarks of human glaucoma including an age-related increase in IOP, and an early insult to RGC axons at the optic nerve head [12-14]. As in humans, lowering IOP lessens or prevents glaucoma in DBA/2J mice [15-17]. Previous studies have shown that activation of the complement cascade occurs in both the retina and the optic nerve head in DBA/2J glaucoma [4,18,19]. Hierarchical clustering of gene expression data predicts that activation of the complement cascade is an early event in DBA/2J glaucoma [4]. Highlighting the importance of the complement cascade, DBA/2J mice mutant for the complement component *C1qa* gene*,* an initiating factor in the classical pathway, show a significant reduction in glaucomatous RGC loss and optic nerve degeneration compared with normal DBA/2J mice [4].

C5 is reported to play both beneficial and damaging roles in neurodegenerative diseases including Alzheimer's disease [20-22] and Huntington's disease [23]. C5 deficiency also delays Wallerian degeneration and inhibits monocyte recruitment after peripheral nerve injury [24]. Therefore, C5 would be predicted to affect glaucoma, but functional experiments to determine its specific role in glaucoma have not been performed. Contributing to the lack of study of C5 in glaucoma is the fact that DBA/2J mice are naturally deficient in C5 [25,26]. Thus, to determine the effects of C5 in glaucoma, we developed C*5-*sufficient DBA/2J mice by transferring a functional *C5* gene from strain C57BL/6J. C5-sufficient DBA/2J mice develop a more severe form of glaucoma than C5-deficient DBA/2J mice indicating an important role for C5 in glaucoma.

## Methods

### Mouse strains, breeding, and husbandry

All experiments were performed in compliance with the ARVO statement for use of animals in ophthalmic and vision research. All experiments involving animals were approved by The Jackson Laboratory Animal Care and Use Committee. All mice were bred at the Jackson Laboratory and housed with a 14-hour-light/10-hour-dark cycle, under the same conditions as previously described [27]. To generate C5-sufficient DBA/2J mice (herein referred to as DBA/2J.*C5*^*B6*^, official strain name DBA/2J.B6-*Hc*^+^/Sj), the interval of mouse chromosome 2 containing a functional *C5* gene was backcrossed to DBA/2J for 11 generations. Mice heterozygous for the B6 region containing *C5* were intercrossed to generate cohorts of DBA/2J mice of all *C5* genotypes (*C5*^*D2/D2*^, *C5*^*D2/B6*^ and *C5*^*B6/B6*^). There were no observable differences between *C5*^*D2/B6*^ and *C5*^*B6/B6*^ mice, thus unless otherwise stated, these are collectively referred to as *C5*^*B6*^.

### Determining the congenic interval in D2.C5^*B6*^ mice

The extent of the B6-derived region was analyzed after 11 generations of backcrossing using polymorphic markers spanning the proximal portion of mouse chromosome 2 (identified using Ensembl and Mouse Genome Informatics). Polymorphic markers included *D2Mit*296 (D2 = 144 bp, B6 = 154 bp), *D2Mit*367 (D2 = 162 bp, B6 = 149 bp), *D2Mit*369 (D2 = 110 bp, B6 = 129 bp), *D2Mit*241 (D2 = 96 bp, B6 = 135 bp) and *D2Mit*376 (D2 = 86 bp, B6 = 92 bp). For each marker, DNA was amplified by PCR from D2.*C5*^*B6*^, D2, and B6 mice under standard conditions. Products were resolved in 3% agarose gels.

### Iris disease and IOP measurement

In standard DBA/2J mice, glaucomatous IOP elevation follows the onset of the iris disease [28]. Therefore, when altering the genotype of DBA/2J mice, it is necessary to evaluate both iris disease and IOP elevation. Iris disease was followed in mice using a slit-lamp biomicroscope as previously reported [29]. IOP measurements were performed using the microneedle method as previously described [30]. Ages and sample sizes are detailed in the results and figure legends. Statistical analysis was performed using ANOVA (JMP statistical package).

### Tissue harvesting and processing

Eyes, with the intra-orbital portion of the optic nerve still attached, were enucleated and fixed overnight in 4% paraformaldehyde (PFA) at 4°C. The retro-orbital portions of the optic nerve (to the chiasm) were carefully removed from the underside of the brain and fixed in 4% PFA overnight. Eyes and optic nerves were stored in 0.5 mol/L phosphate buffer at 4°C.

### Immunofluorescence and RGC quantification

For immunofluorescence, whole retinas were dissected free, washed with 1X PBS-T (phosphate-buffered saline with 0.5% Triton-X100), and incubated with monoclonal antibodies against tubulin β-III (TUBB3; 1:500; Sigma-Aldrich, St Louis, MO, USA) and rabbit polyclonal C5b-9 (1:200; Calbiochem, San Diego, CA, USA) for 96 hours at 4°C. After washing 3 times with PBS-T, the retinas were incubated in the respective secondary antibodies for 2 hours. The retinas were further washed in PBS-T and mounted (Aqua Poly/Mount; Polysciences, Warrington, PA, USA), and imaging was performed (Axio Imager; Zeiss, Jena, Germany). For quantification of TUBB3-immunolabeled RGCs, cuts were made in the retinas to divide it into four equal quadrants, and two digital images (350 × 350 μm) were taken of the peripheral retina in each quadrant. Manual counts were performed using the cell count tool from the Fiji software (http://imagej.nih.gov/ij). The number of retinas per group is given in the Results section and figure legends.

For immunofluorescence of the intra-orbital portion of the optic nerve and optic nerve head, eyes with the intra-orbital nerve still attached were immersed in 30% sucrose (until they sank), rinsed in 1 × PBS and frozen in optical cutting temperature (OCT) compound. Eyes were cut into 10 μm sections, and the central sections containing optic nerve were stained with polyclonal C5b-9 and monoclonal phosphorylated neurofilament (pNF; 1:500, Dako, Glostrop, Denmark) as described previously [4]. For each experiment, at least four sections from six eyes for each glaucoma severity for each genotype were assessed. Imaging and photography was performed on both an imaging microscope (Axio Imager; Zeiss) microscope and a confocal microscope (SP5; Leica, Heerbrugg, Switzerland).

### Assessment of glaucomatous damage

Retro-orbital optic nerves (within 1 to 2 mm of the orbit) were processed for plastic sectioning and *para*-phenylenediamine (PPD) staining to differentiate severity of glaucoma, and analyzed as previously reported [28,31]. Eyes had one of three damage levels: no or early (NOE, <10% axons damaged/lost), moderate (< 50% axons damaged/lost) or severe (> 50% axons damaged/lost).

## Results

### D2.C5^B6^ mice develop a disease of the iris and a glaucomatous IOP profile

**Figure 1:**
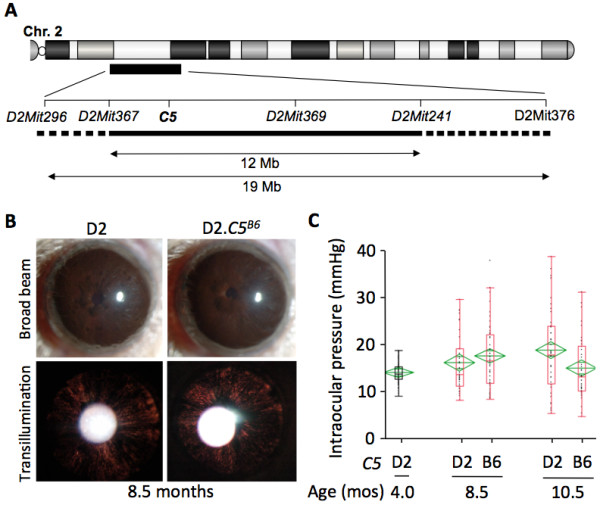
**Progression of iris disease and intraocular pressure (IOP) profiles in DBA/2J and D2.*C5***^***B6 ***^**mice. (A)** The congenic interval surrounding *C5* in D2.*C5*^*B6*^ mice determined using markers polymorphic between D2 and B6 (see Methods). The proximal and distal breakpoints are indicated by the dotted line. The confirmed congenic interval from B6 is shown as a solid line. The size of the congenic interval is between 12 Mb and 19 Mb, which equates to less than 0.5% of the mouse genome. Analysis of known variations between D2 and B6 showed that *C5* is the only gene in this region that has a null mutation in D2 compared with B6. **(B)** Eye examinations with broad-beam illumination (top) and transillumination (bottom) found no obvious differences in iris disease progression between D2.*C5*^*B6*^ and DBA/2J mice. **(C)** Age-dependent elevation of IOP was found in both D2 and D2.*C5*^*B6*^ mice (red) compared with young D2 mice (black). Box plots were generated using JMP version 7.0. The ends of each box represent the 75th and 25th percentile. The lines across the middle of each box indicate the median value. The whiskers extending from either end indicate the extent of the data points. Values falling outside the whiskers are considered outliers. The green diamonds indicate the mean and the 95% confidence interval.

DBA/2J (D2) mice do not express a functional C5 protein, owing to a frameshift mutation in the C5 gene [26]. To determine the affect of a functioning *C5* gene on the progression of D2 glaucoma, the *C5* allele from C57BL/6J (B6) mice was backcrossed into the DBA/2J strain (D2.C5^B6^) (Figure 1A). D2 mice develop a secondary glaucoma as a result of an iris disease, followed by IOP elevation. To determine whether a functioning *C5* gene had an effect on the iris disease, detailed ocular examinations were performed on at least 20 D2 and 20 D2.*C5*^*B6*^ mice at 6, 8, 10, and 12 months of age. Both D2 and D2.*C5*^*B6*^ mice developed the characteristic iris disease (Figure 1B). No obvious differences in the onset or progression of iris stromal atrophy (ISA) or iris pigment dispersion (IPD) were seen between D2 and D2.*C5*^*B6*^ mice (Figure 1B).

We next compared IOP levels between D2.*C5*^*B6*^ and D2 mice (Figure 1C). In D2 mice, IOP first started to increase in a significant number of eyes at 8 to 8.5 months of age and was increased in many eyes at 10.5 months of age [31]. Therefore, IOP levels were measured at these key ages. For both D2.*C5*^*B6*^ and D2 mice, IOP levels were higher than those in young D2 controls. There was no significant difference between the IOP profiles of D2.*C5*^*B6*^ and D2 mice at 8.5 months of age (*P* = 0.23), suggesting that C5 sufficiency does not affect the onset of IOP increase. However, at 10.5 months of age, IOP levels were significantly lower in D2.*C5*^*B6*^ mice compared with D2 mice (*P* = 0.003). This difference suggests that, although *D2.C5*^*B6*^ mice had a glaucomatous IOP profile (as judged by a significant elevation in IOP levels in D2.*C5*^*B6*^ mice at 8.5 (*P* = 0.002) and 10.5 months of age (*P*< 0.0001) compared with DBA/2J control mice at 4.0 months), C5 sufficiency may affect the extent of IOP elevation in D2.*C5*^*B6*^ mice.

### More D2.C5^B6^ mice develop severe glaucoma at an earlier age than D2 mice

**Figure 2:**
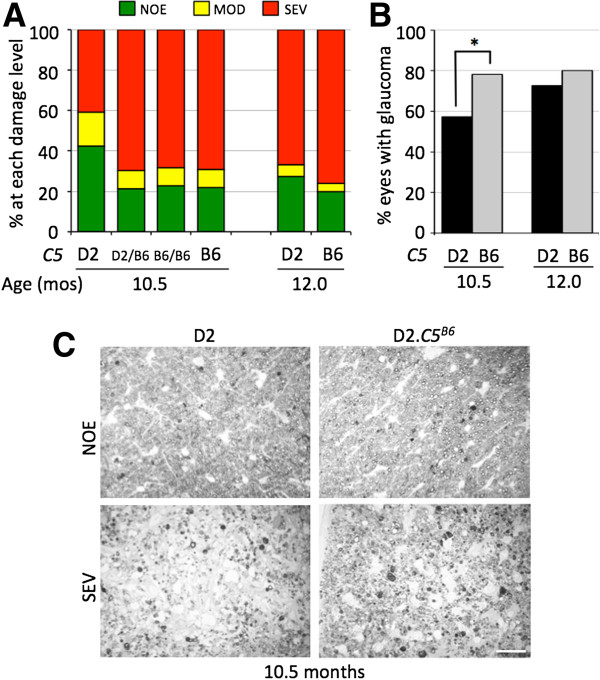
**Glaucomatous neurodegeneration was more severe in D2.*C5***^***B6 ***^**mice. (A)** Distributions of optic nerve damage showed a significant increase in the number of eyes with moderate (MOD; <50% axons damaged/lost) and severe (SEV; >50% axons damaged/lost) glaucoma at 10.5 months in D2.*C5*^*B6*^ compared with normal D2 mice (*P* = 0.0001). There was no difference between D2 mice carrying one (D2.*C5*^*D2/B6*^) or two functioning copies (D2.*C5*^*B6/B6*^) of the *C5* gene. Number of eyes: 10.5 months; D2 = 54, D2.*C5*^*D2/B6*^ = 33, D2.*C5*^*B6/B6*^ = 22, D2.*C5*^*B6*^ (combined D2.*C5*^*D2/B6*^ and D2.*C5*^*B6/B6*^) = 55. 12 months; D2 = 33, B6 (combined) = 25. NOE, no or early glaucoma (no detectable axon loss but some eyes may have early molecular changes). **(B)** A summary of the optic nerve damage assessment shown in **(A)**. At 10.5 months of age, 78% of eyes from D2.*C5*^*B6*^ mice showed either MOD or SEV glaucoma compared with only 57% of D2 mice. **C)** Examples of NOE and SEV damaged nerves in D2 and D2.*C5*^*B6*^. Scale bar = 25 μm.

In our colony, glaucomatous optic nerve degeneration in D2 mice is typically observed between 10 and 12 months of age [1,31]. D2.*C5*^*B6*^ mice showed a significant increase in the number of eyes with severe glaucoma at 10.5 months of age (69%) compared with normal D2 eyes (38%, *P* = 0.0001) (Figure 2). At 12 months of age, the number of D2.*C5*^*B6*^ eyes with severe glaucoma was not significantly different to normal D2 eyes of the same age (76% compared with 67%, *P* = 0.09).

**Figure 3:**
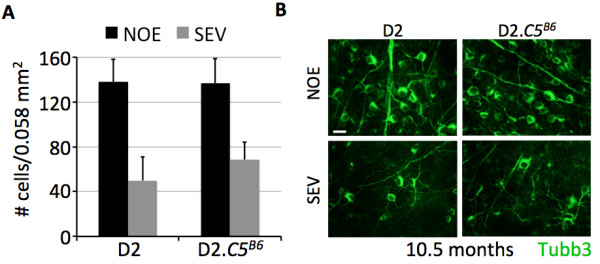
**Significant loss of retinal ganglion cells (RGCs) in both D2 and D2.*C5***^***B6 ***^**mice. (A)** Tubulin β-III (TUBB3)-labeled RGCs were counted in flat-mount retinas with no or early (NOE) or severe (SEV) glaucoma at 10.5 months of age for both DBA/2J and D2.*C5*^*B6*^ mice. RGC degeneration matched the severity of optic-nerve degeneration in both groups. Eight retinas of each age/genotype were counted. **(B)** Example images of TUBB3-labeled RGCs in flat-mount retinas with NOE and SEV glaucomatous damage in D2 and D2.*C5*^*B6*^. Scale bar = 20 μm.

Previous studies have separated RGC axon degeneration in the optic nerve from the death of RGC somas. For instance, in D2 mice that are deficient for the pro-apoptotic gene *BAX*, optic nerves degenerate to the same extent as in normal BAX-sufficient D2 mice, but RGC soma survive [32]. Therefore, to determine the extent of RGC somal loss in D2.*C5*^*B6*^ mice, retinal flat mounts from D2.*C5*^*B6*^ and D2 eyes with no detectable glaucoma (NOE) and severe glaucoma were labeled with TUBB3, and the TUBB3-positive cells in the ganglion cell layer were counted (see Methods). Both D2.*C5*^*B6*^ and D2 eyes with severe glaucoma showed significant loss of RGCs (Figure 3). Collectively, our data showed that a functional *C5* gene significantly exacerbates both optic-nerve degeneration and RGC somal loss in DBA/2J mice.

### MAC deposition on RGC axons and soma very early in glaucoma

**Figure 4:**
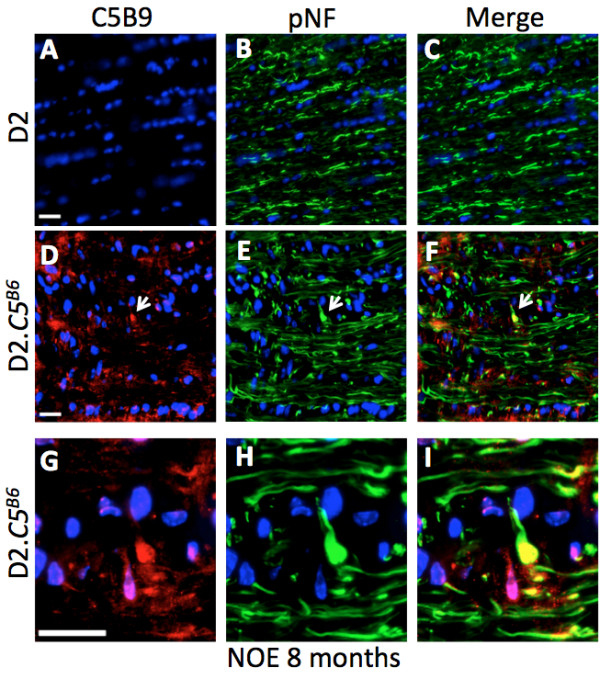
**Membrane attack complex (MAC) deposition in dystrophic axons during early glaucoma in D2.*C5***^***B6 ***^**optic nerves. (A-C)** As expected, no MAC deposition was seen in D2 optic nerve axons at 10.5 months of age. **(D-F)** However, MAC deposition (as judged by the presence of anti-C5b-9) was found in the optic nerve head from D2.*C5*^*B6*^ mice with no or early (NOE) glaucoma. **(G-I)** High magnification views from **D**-**F**, showing some dystrophic neurites were labeled by anti-C5b-9, indicating deposition of MAC in these degenerative structures (arrows). Scale bar = 20 μm.

*C5* has been shown to play multiple roles in neurodegenerative diseases and after neuronal injury [21,23,33-35]. The C5 protein is cleaved by C5 convertases to produce C5a and C5b. C5a plays an important role in inflammation, while C5b is involved in cell lysis [10]. To begin to determine how a functioning C5 protein may contribute to more severe glaucoma in D2.*C5*^*B6*^ mice, optic nerves were analyzed with anti-C5b-9, an antibody that recognizes a functioning MAC. In D2.*C5*^*B6*^ eyes with early glaucoma but no detectable axon loss, MAC deposition was found in RGC axonal swellings (or dystrophic neurites) in the optic nerve (Figure 4). Dystrophic neurites are an early sign of RGC axon damage in the optic nerve head in glaucoma [12]. As expected, C5b-9 was not seen in C5-deficient D2 mice (Figure 4A-C).

**Figure 5:**
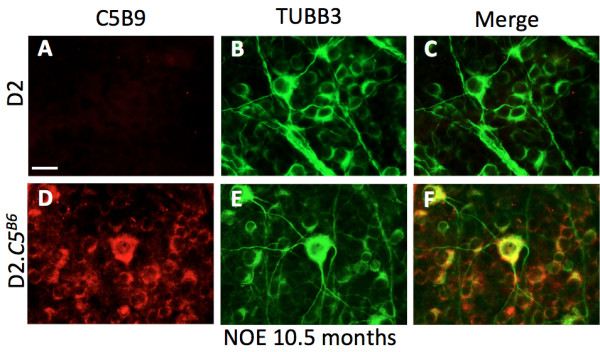
**Membrane attack complex (MAC) deposition in retinal ganglion cell (RGC) soma in D2.*C5***^***B6 ***^**mice. (A-C)** D2 retinas did not show any MAC staining. **(D-F)** Significant deposition of MAC was evident in RGCs from 10.5-month-old D2.*C5*^*B6*^ eyes with no or early (NOE) glaucoma. Scale bar = 20 μm.

Previous work has also shown that the MAC can be deposited on neuronal bodies after injury [20,21,23]. Therefore, to determine whether a functioning *C5* gene results in MAC deposition in RGC soma in glaucoma, we localized C5b-9 in retinas from D2.*C5*^*B6*^ eyes with early glaucoma. To identify RGCs, retinas were co-labeled with anti-TUBB3 antibody. Interestingly, in D2.*C5*^*B6*^ eyes with no detectable optic nerve damage behind the eye, RGCs showed significant levels of MAC deposition (Figure 5). Again, no MAC staining was found in glaucomatous retinas from C5-deficient D2 mice. Together, these experiments support a role for *C5* and the MAC in the early stages of glaucomatous RGC loss in glaucoma.

## Discussion

Previous work has shown increased expression of components of the classical complement pathway in glaucoma [1,4-7,36]. Glaucomatous neurodegeneration was reduced in C1qa-deficient DBA/2J mice, indicating a crucial role of this protein and the complement cascade in general in glaucoma [4]. It is thought that the expression of *C1qa* in either RGCs and/or microglia/monocytes mediates the glaucomatous damage to RGCs, although *C1qa* has also been reported to be expressed in other retinal cell types such as photoreceptors [37]. In this study, we show for the first time that a second key component of the complement cascade, C5, modulates the progression of RGC loss in DBA/2J glaucoma. Our results show that C5 deficiency protects normal D2 mice from glaucoma, with C5 sufficiency rendering mice prone to develop a more severe form of glaucoma.

Similar to other complement proteins, C5 is present in multiple locations and cell types during glaucoma, including the retina and the optic nerve head, and it could contribute to glaucoma in multiple ways including modulation of IOP elevation. For instance, *C1qa* and *C3* have been shown to be necessary for synaptic pruning in the retina during development, and may be necessary for RGC synapse remodeling early in glaucoma [9]. *C1qa* may also mediate ribbon synapse remodeling in photoreceptors in DBA/2J mice [37], although we have not found any photoreceptor changes in our DBA/2J colony [31]. Further work is needed to determine the exact role(s) of C5 in synapse remodeling and other crucial processes in glaucoma.

The C5 protein can be converted into two active peptides, C5a, a proinflammatory anaphylatoxin, and C5b, a key component in the formation of the MAC [10]. Therefore, a functioning *C5* gene may provoke either a proinflammatory effect, the formation of the MAC, or both. C5a is a potent proinflammatory molecule involved in immune-cell recruitment and entry of cells into tissues from the vasculature [38-40], two events we have shown to be important in early stages of glaucoma [41]. It has also been shown that C5a can act directly on neurons through the C5a receptor (*C5aR*) [42,43]. Previous studies have reported that C5a can exert a protective effect on neurons expressing *C5aR* after neuronal excitotoxicity [42,44]. Interestingly, in our microarray studies, *C5aR* was increased in the early stages of glaucoma in both the retina and the optic nerve head [4,45]. However, the C5-sufficient D2 mice were more prone to severe glaucoma compared with the C5-deficient DBA/2J mice, implying either a different role for C5a/C5aR, or a greater, more damaging, contribution from C5b.

Generation of C5b is a necessary step in the formation of the MAC. We found significant MAC deposition in glaucomatous RGCs, and dystrophic neurites in optic nerves of D2.*C5*^*B6*^ mice. MAC deposition in RGCs has also been reported in human glaucoma and in other experimental models [5,46]. Studies have also identified significant MAC deposition in other neurodegenerative diseases such as traumatic brain injury (TBI) [34,47], acute brain ischemia [35], peripheral nerve injury [48], Alzheimer’s disease [20-22,49], and Huntington's disease [23]. Our study suggests that MAC deposition may be a damaging event in glaucoma. This is also supported by studies that show that an increase in MAC deposition (using mice deficient for the MAC inhibitor CD59a) can have adverse neuropathological outcomes in experimental models of TBI [47], peripheral nerve injury [50], and tau pathology [51]. Therefore, although we cannot exclude a contribution of C5a, it is likely that MAC deposition is a major contributor to the increase in glaucoma severity in D2.*C5*^*B6*^ mice. Further work using specific ablation of either C5a or C5b in specific cell types is needed to confirm this.

In summary, we have demonstrated that the downstream components of the complement cascade have a damaging role in glaucoma, and that the natural C5 deficiency protects D2 mice from severe glaucoma. This implies that a functional C5 protein affects RGC loss in human glaucoma, and that inhibition of C5 may be a useful target for treatment. Given that most humans have a functional *C*5 gene, C5-sufficient D2 mice may more closely model human glaucoma, and D2.*C5*^*B6*^ mice are therefore an important new strain for understanding the role of the complement cascade in glaucoma.

## Competing interests

The authors of this manuscript have no competing interests.

## Authors’ contributions

GRH and SWMJ conceived and designed the study, and oversaw all experiments. ISR performed the immunofluorescence experiments and soma counts. MR generated the D2.*C5*^*B6*^ mouse strains, bred the mice, and harvested tissue for characterization. LG determined the congenic interval in the D2.*C5*^*B6*^ strain. RSS performed the characterization of the optic nerves for glaucomatous damage. ISR and GRH wrote the paper, with contributions from SWMJ. All authors have read and approved the final version of the manuscript.
